# Nutritional Composition and Bioactive Compounds of Basil, Thyme and Sage Plant Additives and Their Functionality on Broiler Thigh Meat Quality

**DOI:** 10.3390/foods11081105

**Published:** 2022-04-12

**Authors:** Petru Alexandru Vlaicu, Arabela Elena Untea, Raluca Paula Turcu, Mihaela Saracila, Tatiana Dumitra Panaite, Gabriela Maria Cornescu

**Affiliations:** Nutrition Physiology and Feed and Food Quality Departments, National Research and Development Institute for Biology and Animal Nutrition, 077015 Balotesti, Romania; arabela.untea@ibna.ro (A.E.U.); raluca.turcu@ibna.ro (R.P.T.); mihaela.saracila@ibna.ro (M.S.); tatiana.panaite@ibna.ro (T.D.P.); gabriela_cornescu@yahoo.com (G.M.C.)

**Keywords:** additives, antioxidants, basil, chicken, fatty acids, meat quality, plants, thigh meat, thyme, sage

## Abstract

Meat industries across the world are constantly focusing to find natural low-cost additives for the development of novel meat products to meet consumer demand for improving the health benefits. In this study, we investigated the chemical composition and the bioactive compounds of some herbal plants, namely basil, thyme, sage, and their functionality on broiler chicken thigh meat quality. Chemical composition, as well as total antioxidant activity, polyphenols, vitamin E lutein and zeaxanthin and the fatty acids of the plants, were analyzed. According to findings, total polyphenols was 21.53 mg gallic acid/g in basil, 31.73 mg gallic acid/g in thyme and 38.87 mg gallic acid/g in sage. The antioxidant capacity was 19.91 mM Trolox in basil, 54.09 mM Trolox in thyme and 54.09 mM Trolox in sage. Lutein and zeaxanthin from basil was 267.91 mg/kg, 535.79 mg/kg in thyme and 99.89 mg/kg, and vitamin E ranged from 291.71 mg/kg in basil to 379.37 mg/kg in thyme and 148.07 mg/kg in sage, respectively. After, we developed a trial on 120 unsexed broiler chickens (*n* = 30) which were separated into four groups with six replications of five chickens each: control (C); 1% basil (B); 1% thyme (T) and 1% sage (S). The B, T and S groups deposited significantly higher (*p* < 0.05) concentration of zinc, polyphenols, antioxidant capacity and vitamin E in meat samples compared with the C group. In the experimental groups, the proportion of total polyunsaturated fatty acids, the ratio of n-6 to n-3 fatty acids, and the ratio of polyunsaturated fatty acids to saturated fatty acids in the thigh muscles were significantly improved (*p* < 0.05). The tested plants exhibited a significant (*p* = 0.0007) hypocholesterolemic effect in the meat of the B (45.90 mg/g), T (41.60 mg/g) and S (48.80 mg/kg) experimental groups compared with the C (60.50 mg/g) group. These results support the application of the studied plants as natural sources of additives which could be effective in improving meat quality, from the human consumption perspective.

## 1. Introduction

The worldwide meat industry is constantly evolving due to the requests and changes in consumer preferences. Because of its nutritional composition, chicken meat has played a significant role in human evolution and is an important component of a healthy and well-balanced diet. Part of this evolution is the replacement of synthetic antioxidants with plant-derived ones, due to the public perception that natural compounds are safer and healthier and have the potential to increase meat quality [1]. Beside the fact that chicken meat is a valuable source of high biological nutrients (i.e., protein, vitamins and minerals), the fat content, fatty acid profile, and cholesterol are a constant matter of concern when referring to meat consumption [2].

In order to obtain high-quality meat to meet the consumers’ demands, the best strategy is the utilization of plant-derived supplements as additives in broiler chickens’ diets because they contain numerous bioactive compounds with antioxidant potential. Generally, the most abundant antioxidants in plants are phenolic compounds and vitamins, which play a crucial role in counteracting lipid oxidation in meat [3]. Lipid and protein oxidation in meat are the principal factors that cause meat spoilage, being associated with its intrinsic and extrinsic quality degradation, which further is responsible for general meat quality deterioration [4]. Lastly, meat oxidation is responsible for the formation of some chemical compounds (cytotoxic, mutagenic), thereby promoting chronic diseases, cancer, atherosclerosis, inflammation and aging processes in humans [5].

The history of using plants and spices for human remedies dates back to 5000 Before the Common Era. For this reason, Food and Drug Administration classifies the bioactive substances of some herbal plants and their extracts as generally safe (GRAS) [6]. In some Eastern European countries (Romania, Ukraine, Lithuania, Poland, Belarus, Serbia and Bulgaria), herbal plants (thyme, cloves, cinnamon, oregano, sage, mustard, nutmeg and basil) are very popular and could be obtained for free from nature with no extra costs to affect the feed producers. Therefore, they can be considered one of the first functional food ingredients.

In poultry nutrition, dietary plants as feed additives were successfully used as alternative to antibiotics and health promoting agents, but due to differential results obtained by the scientific community, further research is required [7,8,9]. Bioactive components of herbal plants have high free radical scavenging activity, which might contribute to the endogenous oxidative status of animals and, consequently, might prevent oxidation in the meat, leading to improved meat quality. Moreover, the fatty acid profile in these plants might also enhance broiler meat quality. A review of a series of studies concludes that the inclusion of basil, thyme and sage in broilers diets led to improved productive performance, nutrient digestibility and immune status of animals [10]. Recent experimental evidence supports the benefits attributed to dietary basil [7], thyme [11] and sage [12] plants as feed additives in broiler chickens’ diets. However, because plants also contain tannin, saponin, and other polyphenolic chemicals, an excessive amount of plant supplementation in broilers may have deleterious implications [13].

Given the above-mentioned needs for meat and poultry industry and the modes of action of basil, thyme and sage herbal plants, they represent a possibility to obtain an improved product with functional properties. The scientific proved antioxidant properties of selected plants, coupled with a scarcity of studies following their simultaneous use (*Lamiaceae* family) in chickens’ diets, led us to design a nutritional experiment in order to evaluate their potential for improving the quality of meat. Although the use of herbal plants is very popular for different purposes, we wanted to demonstrate whether the bioactive compounds present in the mentioned plants can produce a functional food of animal origin.

Therefore, in this context, the aim of this study was to determine the effects of dietary basil, thyme and sage as natural feed additives on bioactive compounds with antioxidant potential, fatty acids composition and cholesterol content in thigh meat of chicken broilers.

## 2. Materials and Methods

### 2.1. Plant Materials

Basil (*Ocimum basilicum*), thyme (*Thymus vulgaris*) and sage (*Salvia officinalis*), all members of the *Lamiaceae* family, were bought from the local Medicinal Agency, Plafar (Bucharest, Romania), in dried form. The herbs were powdered in a hammer mill with a 1 mm screen and stored in paper bags at room temperature until the analytical tests were completed, after which they were integrated in compound feeds.

### 2.2. Experimental Design

A total of 120 unsexed Cobb 500 broiler chickens, at the age of 10 days, were distributed into 4 homogeneous groups of 30 chickens with 6 repetitions of 5 chickens in each. They were raised until 42 days of age, when the feeding trial was finished. According to sanitary–veterinary rules (EU 627/15.03.2019), the broiler chicks were housed in an experimental hall with three-tiered Big Dutchman (Vechta, Germany) digestibility cages. The microclimate conditions and the light regimen inside the experimental hall were set at the beginning of the experiment according to the Cobb 500 Breeder Management Guide, and they were automatically monitored with the help of a Viper Touch computer. The main ingredients, corn and soybean meal, were used as a control diet (C) for broilers. Then, in order to formulate isonitrogenous, iso-energetic and isofibrous diets, soybean meal represented the main ingredient from which 1% was replaced by the supplements studied. Three supplemented diets were designated as follows: 1% basil (B), 1% thyme (T), and 1% sage (S), which was made by completely mixing the control diet with the identified supplements at the required incorporation levels. The diets contained no medication and no coccidiostat. Water and feed were available ad libitum. The ingredients of the experimental diets and their chemical composition are described elsewhere [12].

### 2.3. Meat Sample Collection

When the birds were 42 days old, and the feeding trial ended, 24 broiler chickens (6 chickens/group) were selected to be slaughtered according to the procedures presented previously [14]. After dissection and evisceration, the thigh meat without skin was collected and sampled in order to determine the proximate composition, antioxidant compounds, fatty acids profile and cholesterol concentration. The samples were stored in plastic zippered bags at −20 °C until chemical analyses were performed.

### 2.4. Determination of Chemical Composition

The primary chemical composition analysis of the plants and chicken meat was carried out following the methods recommended by the Association of Official Analytical Chemists authorized techniques [15]. The crude protein was determined by the Kjeldahl method (Kjeltec auto 1030 Tecator Instruments, Höganäs, Sweden), the crude fat was determined using a Soxhlet apparatus by extraction in organic solvents (Soxtec 2055 Foss Tecator, Höganäs, Sweden), crude fiber was determined by the method with intermediary filtration (Fibertec 2010 System Foss Tecator, Höganäs, Sweden) and ash content was determined by incineration at 550 ± 15 °C, 3–5 h, until the sample ash became white. The time of calcination depends on tissues’ chemical structure. The analyses of the plants were runed in triplicate and the average value was reported.

### 2.5. Determination of Mineral Composition

Thermo Electron SOLAAR M6 Dual Zeeman Comfort (Cambridge, UK) equipment was used to determine zinc (Zn), iron (Fe), copper (Cu), and manganese (Mn) using atomic absorption spectrometry (FAAS) after microwave digestion, as described by Untea [16]. Following microwave digestion, Thermo Electron SOLAAR M6 Dual Zeeman Comfort (Cambridge, UK) equipment was used to perform atomic absorption spectrometry (FAAS). The obtained results were expressed as mg/kg. The analyses of the plants were runed in triplicate and the average value was reported.

### 2.6. Determination of Total Polyphenols Content and Antioxidant Capacity

The total polyphenol content of plants and meat samples in methanolic extract (1 g dry powder in 10 mL methanol 80%) was determined spectrophotometrically using the Folin–Ciocalteu technique. The calibration curve was made with gallic acid, and the results were represented in milligrams of Gallic acid equivalents per gram of material (mg GAE/g) [17].

The total antioxidant capacity of the extracts was based on the reaction between the sample solution and DPPH reagent prepared in methanol and the absorbance recorded at 517 nm using a V-530 Jasco (Japan Servo Co. Ltd., Tokyo, Japan) spectrophotometer, as described elsewhere [18].

### 2.7. Determination of Vitamin E, Lutein and Zeaxanthin

Vitamin E determination in plants and meat was performed using a high-performance liquid chromatograph (HPLC Finningan Surveyor Plus, Thermo-Electron Corporation, Waltham, MA, USA) and a PDA-UV detector at a wavelength of 292 nm [19].

Lutein and zeaxanthin content were analyzed using a high-performance liquid chromatograph (Perkin Elmer 200 series, Shelton, CT, USA) with a UV detector (445 nm), and a Nucleodur C18 column (Macherey-Nagel, Dueren, Germany), as described here [19]. The results were expressed as mg/kg.

### 2.8. Determination of Fatty Acid and Cholesterol

The fatty acid composition of the plants and meat samples was determined using a gas chromatograph Perkin-Elmer Clarus 500 (Waltham, MA, USA), equipped with a flame ionization detector and capillary separation column with a high polar stationary phase TRACE TR-Fame (Thermo Electron, Waltham, MA, USA), with dimensions of 60 m × 0.25 mm × 0.25 μm, as described elsewhere [20]. The sums and ratios of saturated fatty acids (SFA), monounsaturated fatty acids (MUFA), polyunsaturated fatty acids (PUFA), n-3 fatty acid (n-3) and n-6 fatty acids (n-6) as well as the ratios of PUFA to SFA (PUFA/SFA), n-6 to n-3 (n-3/n-6) and hypocholesterolemic to hypercholesterolemic (H/H) useful for evaluating nutritional value and healthiness of the fatty acid profile were also determined with appropriate formula [21]. The cholesterol concentration was determined by gas chromatography, with the same Perkin-Elmer Clarus according to AOAC [22].

### 2.9. Statistical Analysis

One-way analysis of variance (ANOVA), using Stat View for Windows (SAS, version 6.0, BrainPower Inc., 24009 Ventura Blvd. Suite 250, Calabasas, CA 91302, USA), was carried out to determine the effect of plants on meat quality. Tukey’s multiple range tests were used to determine the significance of individual mean differences. At *p* < 0.05, mean differences were considered significant. The Principal Component Analysis (PCA) was performed to uncover the correlation structure between the analyzed samples using the corresponding function of the Matlab and Simulink (version 2020, MathWorks Inc Bartok B. ut 15/d 1114 Budapest Hungary) software package.

## 3. Results

### 3.1. Nutritional and Chemical Composition of the Plants

The chemical composition of the analyzed plants is shown in Table 1. The proximate composition revealed variable concentrations of crude protein, crude fiber and ash. The obtained data reveal that all analyzed minerals were accumulated by the plants at different concentration.

**Figure 1:**
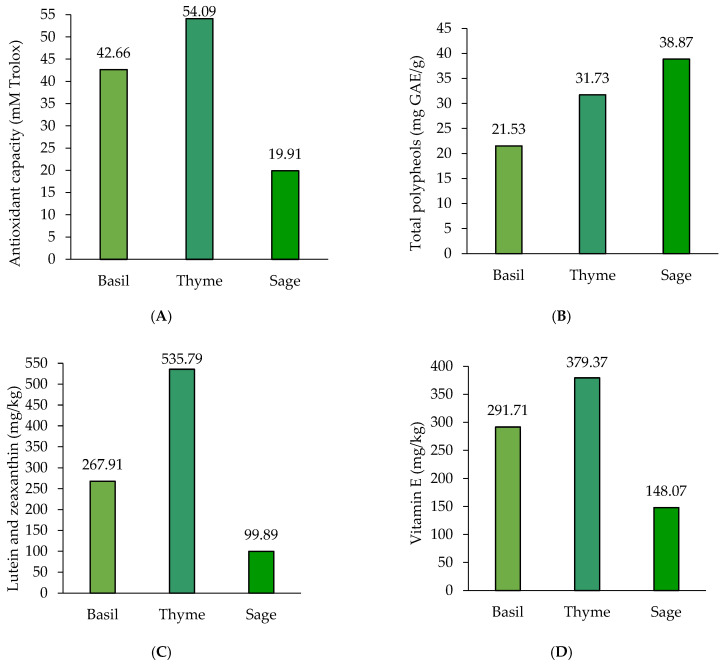
Bioactive compounds with antioxidant activity of plants; (**A**) total antioxidant capacity; (**B**) polyphenol content; (**C**) lutein and zeaxanthin; (**D**) vitamin E content; (*n* = 3).

The antioxidant compounds determined in the plants showed variable results. The antioxidant capacity in basil was with 21.13% lower than in thyme, but with 63.19% higher than in sage (Figure 1A). The total polyphenols content in sage was with 44.61% higher than in basil and with 18.36% than in thyme (Figure 1B). Lutein and zeaxanthin content in thyme was noted to be higher than in basil and sage (Figure 1C). The same result was also observed for vitamin E content in thyme as being higher than in basil and sage (Figure 1D). As it can be noticed, thyme presented the highest number of liposoluble antioxidants, and even if it cannot be considered a valuable source of polyphenols, thyme registered the most important antioxidant capacity.

The fatty acid composition of the dietary plants was also analyzed and is presented in Table 2. The highest concentration of total SFA was noted in thyme. Basil presented the highest concentration of total MUFA, with oleic acid, as dominant in all three plants. It was noted that from the total PUFA, sage presented high concentration of n-6 PUFA, especially linoleic and arachidonic acids. Thyme was noted to be rich in n-3 PUFA, from which α-linolenic was the most abundant. However, basil had the closest n-6/n-3 ratio to the ideal value of 1.

### 3.2. Effect of Dietary Plants on Chemical Composition of Chicken Thigh Meat

As reported in Table 3, feeding diets supplemented with different plants did not lead to any modifications among the groups, regarding the chemical composition of meat. From the mineral composition of meat obtained from chickens fed dietary basil, thyme and sage, it was observed that zinc occurred at a higher concentration (*p* < 0.05) in B, T and S chicken meat samples than in the C samples followed by iron.

### 3.3. Effect of Dietary Plants on Antioxidant Activity in Chickens Thigh Meat

**Figure 2:**
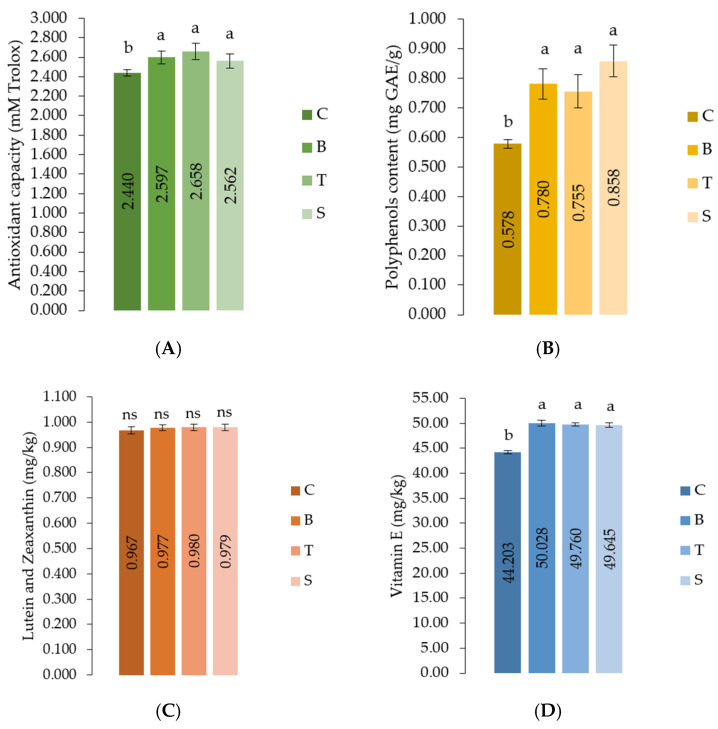
Effect of dietary plant on concentrations of compounds with biological value and antioxidant potential, determined in meat samples; (**A**) total antioxidant capacity; (**B**) total polyphenol content; (**C**) lutein and zeaxanthin; (**D**) vitamin E; ns—not significant; ^a,b^ Different superscript letters are significantly different by Tukey’s multiple comparison method (*p* < 0.05); C- the control diet; B—a diet containing 1% basil; T—a diet containing 1% thyme; S—diet containing 1% sage; (*n* = 6).

Among the compounds determined in the thigh meat samples with biological value and antioxidant potential (Figure 2), the highest increase (*p* < 0.05) of total polyphenol content (Figure 2A), antioxidant capacity (Figure 2B) and vitamin E (Figure 2C) was observed in all three experimental groups that included basil, thyme and sage. No effect was noted for lutein and zeaxanthin (*p* = 0.1634) concentration (Figure 2D).

### 3.4. Effect of Dietary Plants on Fatty Acids Profile of Thigh Meat

The use of dietary plants leads to significant changes in the total SFA content (Table 4). In fact, it was observed that the concentration of butyric, caproic, caprylic, capric, myristic, pentadecanoic and palmitic, fatty acids were significantly lowered (*p* < 0.05) in the B, T and S meat samples, compared with the C samples. However, the significant variations were noted between the C and the S group (*p* = 0.0009). From the total MUFA, significantly (*p* = 0.0001) lower concentrations of palmitoleic acid were determined in the B and S samples compared with the C and T samples, while the concentrations of miristoleic, oleic and erucic fatty acids were significantly (*p* < 0.05) lower only in the S samples compared to all the other groups. However, the basil group had the highest concentration of stearic acid while the sage had the highest concentration of lignoceric acid compared with the other groups. In contrast, in the thyme and sage samples, an increase in total n-6 PUFA (*p* < 0.05) was observed compared to the control and basil samples. The thyme and sage supplemented groups presented significantly higher (*p* < 0.05) concentration of linoleic, arachidonic and docosadienoic acids. Finally, the use of basil, thyme and sage significantly (*p* < 0.05) increased the content of total n-3 PUFA compared to control group. From this group, the results showed that concentration of α-linolenic (ALA), eicosapentaenoic (EPA) and docosahexaenoic (DHA) fatty acids determined in the B, T and S samples was significantly (*p* < 0.05) increased compared with the C samples. Overall, the total PUFA was significantly (*p* = 0.0023) higher in experimental samples while the n-6/n-3 ratio was significantly (*p* = 0.0310) lower. Further, there was a significant reduction (*p* = 0.0007) in cholesterol concentration of chicken thigh meat from birds fed diet supplemented with plants compared with control group.

### 3.5. Principal Component Analysis (PCA)

**Figure 3:**
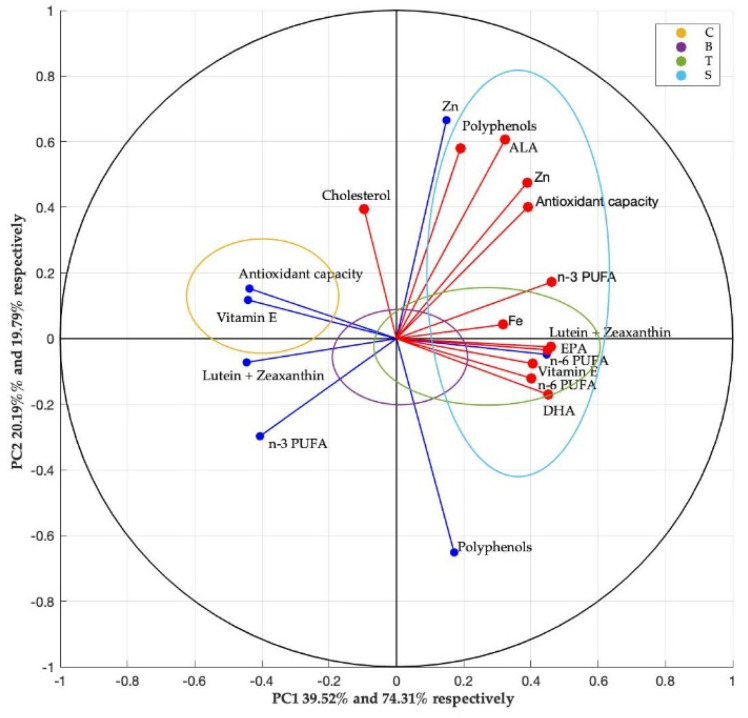
Biplot correlation circle from principal component (PC) analysis of plants (blue lines) and meat (red lines). PC1 covered 39.52% of variance in plants and 74.31% variance in meat. PC2 covered 20.19% variance in plants and 19.79% variance in meat.

PCA is a multivariate approach that is frequently used to reduce data dimensionality. The application of PCA enabled for easier analysis and comparison of similarities between groups by lowering the number of variables. We considered a cantered and normalized version of the data to obtain the PCA representation (Figure 3). The first component (PC1) covered 39.52% and 74.31%, respectively, of the global variance of the data obtained for plants and meat while the second component (PC2) covered about 20.19% and 19.79% of the global variance, respectively.

## 4. Discussion

### 4.1. Nutritional and Chemical Composition of Basil, Thyme and Sage Plants

The nutritional and chemical composition showed a large variation between the studied plants for crude protein in basil, thyme and sage. It was also noted that basil and thyme had lower content of crude fibre compared with sage. Copper concentration was more than three times higher in basil compared with thyme and sage. Iron concentration in sage was higher compared with basil and thyme, while the manganese concentration was higher in thyme, and zinc concentration was higher in basil. Both thyme and sage were noted to present high polyphenols content compared with basil, while on the other hand, basil and thyme had higher antioxidant capacity compared to sage. Further, basil and thyme presented higher lutein and zeaxanthin than sage as well as vitamin E. From the fatty acids, which generally are the main components in oilseeds with beneficial effect for human health, sage was characterized by lower content of total SFA compared with basil and thyme. However, all three plants (basil, thyme and sage) presented considerable concentrations of total PUFA with α-linolenic acid as leading essential n-3 PUFA and linolenic acid as dominant n-6 PUFA. Literature data revealed similar or contradictory results regarding the nutritional composition of these studied plants. For instance, [23] reported similar crude protein (22.08%) in basil, but with higher crude fibre content (25.52%). Thyme reported a lower crude protein (5.23%) and higher crude fibre (18.10%) content [24]. A very low concentration of crude protein in sage (1.3%) was reported [25], but the content of crude fibre (31%) was closer to our determined value (Table 1). Lower concentrations of iron, zinc, manganese, and copper among different cultivars of basil, thyme and sage were reported in other studies [25,26,27,28] compared with our data. Other researchers [29], after studying the antioxidant activity in fifteen species of basil, reported major variations for antioxidant capacity (4.2 to 19.5%) and polyphenol content (2.59 to 8.26 g GAE/g). Moreover, [30] reported higher polyphenols content in sage when compared with thyme. Recently, [31] determined the nutritional composition in nine herbal plants and revealed that basil, thyme and sage presented significant content of polyphenols (11.37; 34.13 and 50.20 mg GAE/g respectively) as well as vitamin E (113.3; 118.93 and 160.76 mg/kg, respectively). Compared with sea buckthorn [32,33] or grapeseed [34], which are natural oilseed byproducts rich in antioxidant compounds, we determined higher concentrations of some essential fatty acids in the studies plants. These results conclude the fact that plant cultivars vary in their nutrient concentrations. This variability in chemical composition, minerals, antioxidant compounds and fatty acids can be attributed to the moment of harvest, climate genotype, storage conditions, temperature, light, soil type and other conditions, which further could lead to different results when tested on chickens’ meat quality.

### 4.2. Effect of Dietary Plants on Chemical and Mineral Composition of Chicken Thigh Meat

In the present study, dietary plants did not influence the chemical compositions of thigh meat. The crude protein concentration was 1.47% lower in T samples, 0.81% lower in B samples and 0.54% in S samples compared to C samples (*p* = 0.9938). The content of crude fat in the C samples was slightly higher (8.36%), compared with that determined in the B (7.87%), T (8.08%) and the S (7.88%) samples (*p* = 0.0671). For the ash content in the thigh meat samples, the results were similar among the groups (*p* = 0.8591). These results agree with other recent observations [35,36,37] that found no significant differences in dry matter, crude protein, fat, and ash contents of broiler meat when using different dietary plants in the broiler diets. The diverse composition of the plants is reflected in a complex influence on bioaccumulation of essential minerals for human health in chicken thigh meat. The only mineral which was significantly (*p* = 0.0408) influenced by dietary plants was zinc. Compared with the C samples, the concentration of zinc in the B samples was with 13.48% higher, and in the T and S samples, it was 7.81% and 7.59% higher, respectively. The thigh portion of the broilers also had a slight, but not significantly higher, amount of iron concentration present. These results are in accordance with other authors who reported that different feed additives (i.e., sage, oregano, anise, citrus fruits, or small-flowered willowherb) increased the concentration of zinc and iron in chicken meat [38,39]. However, each plant had a specific influence on the accumulation of minerals, as in the case of the B samples which presented the highest accumulation of zinc, as reported in Table 3. This could be caused by the antagonism between minerals ions and presence of other chelating agents from the feed of chickens which can act as competitor for mineral complexation and influence accumulation of these trace elements in chicken meat [39]. Despite substantial research into zinc-dependent biochemical pathways in physiologic functioning, definite correlations have yet to be discovered. In contrast to iron, which is found in certain cellular components and has specific physiological functions, zinc is found throughout cells. Nevertheless, as it was reported by Food Agricultural Organization [40], the increased bioavailability of certain minerals like zinc and iron in meat is also beneficial for human health. Generally, human zinc intakes range from 14 to 30 mg/kg/day. These intakes support the zinc balance in healthy adults, but balance can be achieved when as little as 2.8 mg/kg/day or as much as 40 mg/kg/day is fed. However, on the other hand, phytic acid has been shown to be the main dietary component that limits zinc bioavailability by tightly binding zinc in the gastrointestinal tract [41]. Also, zinc is considered a powerful indirect antioxidant compound, being very efficient in prevention of free radical formation and in the retarding of oxidative processes [42].

### 4.3. Effect of Plants on Antioxidant Compounds of Chicken Meat

In the present study, dietary basil, thyme and sage significantly improved the bioactive compounds with antioxidant potential in thigh meat (Figure 2). The increased antioxidant capacity in meat is mainly related to the presence of phenolic compounds in plants. Polyphenol’s content was significantly increase in thigh meat as a response of the additives (Figure 2B), especially in the S group. This is a beneficial effect in terms of impact on human health because they exert immunomodulatory, anti-inflammatory, antioxidant, antimicrobial, antimutagenic, antiallergic and detoxification activities [43]. Moreover, antioxidants are essential for maintaining maximum health and wellness as they protect us from free radical damage. It has been suggested that an intake of a rich antioxidant food could prevent the risk of cardiovascular disease [44]. Exogenous antioxidant consumption from plant, animal, and mineral sources has been shown to improve human health and minimize the occurrence of free-radical-induced illnesses generation. They have also been linked an improvement in antioxidant status in patients, suggesting that they may be useful in regaining normal function and treating such disorders [45]. The significant increase in vitamin E (α-tocopherol) concentration in chicken meat is another effective way to improve the antioxidant content in meat and the oxidative stability of unsaturated fatty acids. Within the cell membrane, where it prevents membrane fatty acids from lipid peroxidation, vitamin E is a crucial lipid-soluble and a highly potent chain-breaking antioxidant. Thus, increasing antioxidant capacity in meat could decrease the formation of reactive oxygen and nitrogen species which are responsible for the development of oxidative stress [46]. However, despite these benefits, numerous studies have investigated the effectiveness of some plants as natural additives rich in antioxidant compounds in poultry meat, but their results are inconsistent. Similar to our results, some studies reported that rosemary [47], oregano, cinnamon [48,49], sage [50], thyme, cumin, basil, garlic and pepper [51] addition to broiler meat had the potential to increase the antioxidant activity in different ways. In contrast, others [52,53], reported that herbal feed additives, or their extracts, did not exert antioxidant activity on chickens’ meat. The synergistic or antagonistic effects between main antioxidant components and other compounds found in the plants could be the explanation which led to these differential results. Nevertheless, because the antioxidant content of meat is often low, supplementing broiler chickens’ diets with herbal plants, spices, fruits, and plant-based foods may still be key sources of our antioxidant intake.

### 4.4. Effect of Plants on Fatty Acid Composition and Cholesterol Content of Chicken Meat

The incorporation of n-3 PUFA and essential fatty acids such as linoleic, linolenic, and arachidonic fatty acids in the diet can play a natural preventive role in cardiovascular disease and other health problems [21]. In this context, dietary basil, thyme and sage supplementation significantly improved the thigh meat fatty acid profiles because of the increases in the health-promoting ALA, EPA, DHA and total PUFA contents. A mean increase in the ALA content by 62.5%, 61.7% and 72.72%, respectively, was determined between the plant supplemented groups and the control group. Moreover, the concentration of EPA was significantly higher, but the DHA was more than double in experimental meat samples compared with control samples. As a result, adding plants to broiler diets improved not only the antioxidant characteristics of the thigh meat but also its nutritional value by fortifying it with health-promoting n-3 PUFA. However, because no PUFA variations were identified between the experimental diets, the higher PUFA content in the thigh meat of broiler chickens fed with natural feed additives could be attributable to the antioxidants’ protective impact on PUFA from oxidative breakdown. Beside the numerous studies regarding the use of different oilseeds, algae or fish oils, which are already demonstrated to be capable to increase the content of these beneficial nutrients [21,54], our results clearly show that some added plants as natural additives are capable to do the same. In agreement with our results, the ALA and PUFA contents were increased as a response to dietary citrus pulp as feed additives [55,56], but a decrease in MUFA and SFA with no significant effect on total n-3 fatty acids was observed. On the contrary, [57] reported that 1% mixture of plants and probiotics decreased the levels of DHA and PUFA in chicken meat, but the use of 0.5% water plantain increased the PUFA and total n-3 fatty acids. Further, lower PUFA/SFA and higher n-6/n-3 are considered undesirable because they may increase cholesterolemia [58,59]. In this experiment the n-6/n-3 ratio significantly (*p* = 0.0310) decreased in the experimental thigh meat, indicating a beneficial effect of plants on broiler meat quality. Dietary plants also increased the PUFA/SFA ratio, although no significant differences were observed for these indices. These results are in line with [60,61] but in contrast with others [57], who failed to increase the content of PUFA in chickens’ meat. However, it has been reported that the PUFA/SFA ratio is not always adequate to evaluate the nutritional quality of the fat. For that reason, the utilization of the ratio between hypocholesterolemic/hypercholesterolemic fatty acid (H/H) is a better approach to evaluate the nutritional quality of the fat [62]. The higher values obtained for this ratio in the thigh meat of chickens was with 4.04%, 7.26% and 11.7%, respectively, in the B, T and S samples, indicating a positive effect of dietary plants on broiler meat quality.

The modulation of the fatty acid composition in meat samples as well as of the H/H ratio is also reflected in the cholesterol concentration. The cholesterol content of thigh meat was significantly (*p* = 0.0007) lowered in chickens fed plant supplemented diets. It was reported that in general, raw poultry meat has approximately 27 to 90 mg cholesterol/100 g [63]. In the present study, we observed that the plant additives had a great implication in the reduction of cholesterol concentration in thigh muscle. Compared to the above-mentioned maximum limit, we obtained a decrement with 53.77% in the B samples, with 49% in the T samples and with 46.22% in the S samples. Similarly, previous works showed that different feed additives significantly lowered the cholesterol concentration in chicken meat [64,65,66,67]. However, we found that not all experimental trials succeeded when different feed additives were tested on meat quality. Some authors [68,69] reported that natural feed additives showed no effect on cholesterol content and had negative impact on meat quality of broiler chickens. We believe that this reduction was caused by the total SFA determined in the tested plants, regarded as having great implications in cholesterol reduction. Beside the effect of SFA, cholesterol reduction may also be caused by the sterols which naturally occur in herbal plants. They have a great role in competing for the absorption of dietary cholesterol and inhibit the re-absorption of endogenous cholesterol in the intestinal tract of animals as it was found previously [70]. This further resulted in a reduced cholesterol concentration in meat. Nevertheless, the implications of natural additives in the reduction of cholesterol in meat deserves further attention.

### 4.5. Principal Component Analyses

The principal component analysis (PCA) was conducted in order to explore the relationship between bioactive compounds in plants and nutrients determined in meat samples (Figure 3). PCA on these attributes explained 59.71% of the variability in the data of the plants and 94.1% in the data of meat samples in the first two dimensions. The loading of PC1 in plants had a strong positive correlation with vitamin E, antioxidant activity, lutein and zeaxanthin and n-6 PUFA. The strong positive correlation of PC2 was between zinc and polyphenols in plants. For meat samples, the score of the PC1 was primarily occupied by iron, EPA, lutein and zeaxanthin, vitamin E, n-6 PUFA, n-3 PUFA and DHA. In the PC2, the strongest correlation was observed for polyphenols, cholesterol, ALA, zinc and lastly antioxidant capacity. In the plotted loadings of PC1, the compounds with antioxidant potential determined in plants and total n-3 PUFA were strongly correlated with the essential fatty acids from meat samples (ALA, EPA and DHA), iron, vitamin E and lutein and zeaxanthin. These variables are positioned in opposite directions alongside the axis of PC1. Further, the biplot of PC2 showed that there was strong correlation between polyphenols and zinc from plants with the cholesterol, polyphenols, ALA and zinc from meat samples. The four groups were sorted roughly and utilized to create the image in biplots (combined score and loading plots). This diagram depicted how these four groups were formed differently depending on their chemical composition. The S group was notably differed on the first and fourth dimensions, occupying nearly all nutrients in meat. Second dimension was dominated by the C group, which clearly does not have any correlation with meat samples. Third and fourth dimension was occupied by the B group, with slight intersection on first and second dimension. Lastly, the T group was dominant in fourth and first dimensions, presenting the strong correlation among all the variables. Overall, it is clear that the three experimental meat samples are intersecting with each other, but they are differentiated compared to the C group.

## 5. Conclusions

The results of the study proved that the dietary inclusion of selected plants on broilers diets exhibited a positive effect on antioxidant compounds and lipid quality of meat. Moreover, basil, thyme and sage through their antioxidant potential can provide a nutritional way of developing functional food of animal origin.

## Figures and Tables

**Table 1:** Nutritional and mineral composition of the plants.

Item	Basil	Thyme	Sage
Chemical Composition *			
Dry matter, %	91.35	91.65	90.64
Crude protein, %	22.53	15.38	9.56
Crude fat, %	1.51	2.09	3.15
Crude fibre, %	12.22	17.08	27.92
Ash, %	14.12	9.43	10.36
Mineral Composition *			
Copper, mg/kg	27.69	7.41	7.89
Iron, mg/kg	624.51	690.05	732.72
Manganese, mg/kg	78.46	96.11	68.92
Zinc, mg/kg	54.63	31.73	38.87

* The values are reported as average of three determinations (*n* = 3).

**Table 2:** Fatty acid composition of the plants.

Fatty Acids, g/100 g	Basil	Thyme	Sage
Caproic C6:0	0.48	0.36	1.72
Caprylic C8:0	0.45	0.38	6.88
Capric C10:0	0.75	0.51	0.39
Lauric C12:0	1.92	0.94	2.38
Myristic C14:0	5.20	21.41	0.43
Pentadecanoic C15:0	0.59	nd	0.38
Palmitic C16:0	22.98	17.12	21.38
Heptadecanoic C17:0	nd	0.08	0.49
Stearic C18:0	8.16	3.06	4.10
Tricosanoic C23:0	0.00	0.00	0.64
**SFA**	**40.52**	**43.86**	**38.79**
Miristioleic C14:1	0.73	1.59	0.27
Pentadecenoic C15:1	1.41	1.23	3.23
Palmitoleic C16:1	1.99	0.89	2.46
Heptadecenoic C17:1	nd	0.06	0.29
Oleic cis C18:1	17.85	7.54	12.65
Nervonic C24:1n9	0.00	0.68	0.80
**MUFA**	**21.99**	**11.98**	**19.70**
Linoleic cis C18:2n6	17.36	12.62	11.40
Linolenic γ C18:3n6	nd	0.16	nd
Eicosadienoic C20:2n6	nd	0.17	3.21
Eicosatrienoic C20:3n6	nd	nd	3.48
Arachidonic C20:4n6	0.55	0.46	5.00
**n-6 PUFA**	**17.91**	**13.41**	**23.08**
α-Linolenic C18:3n3	15.95	27.96	12.61
Octadecatetraenoic C18:4n3	2.71	0.90	5.27
Eicosapentaenoic C20:5n3	nd	0.92	nd
**n-3 PUFA**	**18.66**	**29.78**	**17.87**
**PUFA**	**36.57**	**43.19**	**40.96**
Others	0.92	0.97	0.56
n-6/n-3 ratio	0.96	0.45	1.29

SFA—saturated fatty acids; MUFA—monounsaturated fatty acids; PUFA—polyunsaturated fatty acids; nd—not determined; The values are reported as average of three determinations (*n* = 3).

**Table 3:** Effect of dietary plants on chemical and mineral composition of chicken meat.

Item	C	B	T	S	SEM	*p*
Chemical Composition						
Dry matter, %	28.23	29.53	28.08	28.78	0.549	0.6568
Crude protein, %	18.33	18.18	18.06	18.23	0.321	0.9938
Crude fat, %	8.36	7.87	8.08	7.88	0.291	0.0671
Ash, %	1.11	1.15	1.14	1.06	0.023	0.8591
Mineral Composition						
Copper, mg/kg	1.10	1.27	1.07	1.14	0.058	0.8592
Iron, mg/kg	38.14	41.00	41.28	41.19	0.723	0.2592
Manganese, mg/kg	0.08	0.11	0.09	0.10	0.022	0.1056
Zinc, mg/kg	50.63 ^b^	58.52 ^a^	54.92 ^a^	54.79 ^a^	1.075	0.0408

^a,b^ Means in the same row with different superscript letters are significantly different by Tukey’s multiple comparison method (*p* < 0.05). C—control diet; B—a diet containing 1% basil; T—a diet containing 1% thyme; S—diet containing 1% sage; (*n* = 6); SEM = standard error of the mean.

**Table 4:** Effect of dietary plants on fatty acid profiles (g/100 g) of thigh meat.

Fatty Acids, g/100 g	C	B	T	S	SEM	*p*
Butyric C4:0	0.170 ^a^	0.123 ^b^	0.121 ^b^	0.090 ^c^	0.007	<0.0001
Caproic C6:0	0.122 ^a^	0.102 ^b^	0.107 ^b^	0.083 ^b^	0.004	0.0018
Caprylic C8:0	0.352 ^a^	0.138 ^b^	0.063 ^c^	0.073 ^c^	0.021	<0.0001
Capric C10:0	0.288 ^a^	0.113 ^b^	0.093 ^bc^	0.048 ^c^	0.019	<0.0001
Lauric C12:0	0.04	0.03	0.03	0.03	0.003	0.6454
Myristic C14:0	1.040 ^a^	0.720 ^b^	0.623 ^b^	0.528 ^c^	0.042	<0.0001
Pentadecanoic C15:0	0.455 ^a^	0.327 ^b^	0.378 ^b^	0.367 ^b^	0.013	0.0336
Palmitic C16:0	27.07 ^a^	26.37 ^a^	26.12 ^a^	23.13 ^b^	0.333	0.0002
Heptadecanoic C17:0	0.08	0.27	0.18	0.17	0.023	0.0948
Stearic C18:0	7.652 ^b^	8.705 ^a^	7.878 ^b^	8.095 ^b^	0.109	0.0009
Lignoceric C24:0	0.563 ^b^	0.530 ^b^	0.637 ^ab^	0.697 ^a^	0.020	0.0280
**SFA**	**37.84 ^a^**	**37.428 ^a^**	**36.227 ^a^**	**33.312 ^b^**	**0.411**	**0.0009**
Miristoleic C14:1	0.278 ^a^	0.232 ^ab^	0.242 ^a^	0.153 ^b^	0.083	0.0287
Pentadecenoic C15:1	1.59	1.84	1.48	1.18	0.075	0.0678
Palmitoleic C16:1	5.203 ^a^	4.157 ^b^	4.983 ^ab^	3.643 ^c^	0.117	<0.0001
Heptadecenoic C17:1	0.23	0.28	0.28	0.24	0.018	0.5986
Oleic cis C18:1n9	40.24 ^a^	39.62 ^a^	39.75 ^a^	35.86 ^b^	0.460	0.0040
Erucic C22:1n9	2.695 ^a^	3.222 ^a^	2.663 ^a^	1.708 ^b^	0.142	0.0032
Nervonic C24:1n9	0.092 ^b^	0.057 ^b^	0.092 ^b^	0.235 ^a^	0.018	0.0046
**MUFA**	**50.32 ^a^**	**49.40 ^a^**	**49.49 ^a^**	**43.03 ^b^**	**0.713**	**0.0007**
Linoleic cis (LA) C18:2n6	5.695 ^b^	5.950 ^b^	7.193 ^a^	7.150 ^a^	0.201	0.0482
Linolenic γ C18:3n6	0.09	0.05	0.11	0.07	0.012	0.7736
Conjugated LA C18:2	0.50	0.52	0.42	0.31	0.026	0.0782
Eicosadienoic C20:2n6	0.28	0.32	0.24	0.24	0.013	0.2434
Eicosatrienoic C20:3n6	0.12	0.17	0.11	0.14	0.008	0.1917
Arachidonic C20:4n6	0.092 ^b^	0.110 ^b^	0.103 ^b^	0.820 ^a^	0.083	0.0088
Docosadienoic C22:2n6	0.327 ^b^	0.373 ^b^	0.485 ^a^	0.465 ^a^	0.015	0.0001
Docosatrienoic C22:3n6	0.37	0.43	0.51	0.45	0.017	0.1213
Docosatetraenoic C22:4n6	0.22	0.16	0.31	0.20	0.032	0.4246
**n-6 PUFA**	**7.617 ^b^**	**8.062 ^b^**	**9.445 ^a^**	**9.623 ^a^**	**0.223**	**0.0025**
α-Linolenic (ALA) C18:3n3	0.090 ^c^	0.240 ^b^	0.263 ^b^	0.333 ^a^	0.021	0.0350
Octadecatetraenoic C18:4n3	0.81	0.89	0.67	0.59	0.043	0.1228
Eicosatrienoic C20:3n3	0.058 ^b^	0.080 ^b^	0.221 ^a^	0.230 ^a^	0.017	0.0032
Eicosapentaenoic (EPA) C20:5n3	0.443 ^b^	0.523 ^a^	0.533 ^a^	0.557 ^a^	0.015	0.0081
Docosapentaenoic C22:5n3	0.093 ^b^	0.223 ^a^	0.340 ^a^	0.390 ^a^	0.045	0.0452
Docosahexaenoic (DHA) C22:6n3	0.130 ^b^	0.332 ^a^	0.372 ^a^	0.350 ^a^	0.031	0.0160
**n-3 PUFA**	**1.614 ^b^**	**2.283 ^a^**	**2.394 ^a^**	**2.450 ^a^**	**0.068**	**<0.0001**
PUFA	9.240 ^b^	10.35 ^a^	11.72 ^a^	12.07 ^a^	0.261	0.0023
others	2.621 ^b^	2.823 ^a^	2.718 ^a^	2.020 ^b^	0.141	0.0189
n-6/n-3 ratio	4.719 ^a^	3.531 ^b^	3.945 ^b^	3.927 ^b^	0.164	0.0310
PUFA/SFA	0.24	0.28	0.32	0.36	0.144	0.0615
Cholesterol, mg/100g DM	60.50 ^a^	45.90 ^b^	41.60 ^b^	48.40 ^b^	0.002	0.0007
H/H	1.66 ^b^	1.73 ^a^	1.79 ^a^	1.88 ^a^	0.033	0.0302

^a,b,c^ Values with different superscripts in the same row differ significantly (*p* < 0.05). C—control diet; B—a diet containing 1% basil; T—a diet containing 1% thyme; S—diet containing 1% sage; SEM—standard error of the mean; SFA—saturated fatty acids; MUFA—monounsaturated fatty acids; PUFA—polyunsaturated fatty acids; PUFA/SFA—polyunsaturated to saturated fatty acids ratio; HH—ratio between hypocholesterolemic/hypercholesterolemic fatty acid; (*n* = 6).

## Data Availability

Data is contained within the article.
